# Validity of the Paediatric Canadian Triage Acuity Scale in a Tertiary Hospital: An Analysis of Severity Markers' Variability

**DOI:** 10.1007/s10916-023-01913-8

**Published:** 2023-01-30

**Authors:** João Viana, Raquel Bragança, João Vasco Santos, Alexandra Alves, Almeida Santos, Alberto Freitas

**Affiliations:** 1 CINTESIS – Centre for Health Technology and Services Research, Faculty of Medicine, University of Porto, Porto, Portugal; 2 MEDCIDS – Department of Community Medicine, Information and Health Decision Sciences, Faculty of Medicine of the University of Porto, Al. Prof. Hernâni Monteiro, 4200-319 Porto, Portugal; 3 Serviço de Pediatria / Urgência Pediátrica, UAG da Mulher e da Criança, Centro Hospitalar Universitário de São João, Porto, Portugal; 4 Public Health Unit, ACES Grande Porto V-Porto Ocidental, ARS Norte, Porto, Portugal; 5 Departamento de Ginecologia-Obstetrícia e Pediatria, Faculty of Medicine of the University of Porto, Porto, Portugal

**Keywords:** Children, Triage, Emergency Services, Validity, Sensitivity, Specificity, Likelihood ratios

## Abstract

With the increasing influx of patients and frequent overcrowding, the adoption of a valid triage system, capable of distinguishing patients who need urgent care, from those who can wait safely is paramount. Hence, the aim of this study is to evaluate the validity of the Paediatric Canadian Triage and Acuity Scale (PaedCTAS) in a Portuguese tertiary hospital. Furthermore, we aim to study the performance and appropriateness of the different surrogate severity markers to validate triage. This is a retrospective study considering all visits to the hospital’s Paediatric Emergency Department (PED) between 2014 and 2019. This study considers cut-offs on all triage levels for dichotomization in order to calculate validity measures e.g. sensitivity, specificity and likelihood ratios, ROC curves; using hospital admission, admission to intensive care and the use of resources as outcomes/markers of severity. Over the study period there were 0.2% visits triaged as Level 1, 5.7% as Level 2, 39.4% as Level 3, 50.5% as Level 4, 4.2% as Level 5, from a total of 452,815 PED visits. The area under ROC curve was 0.96, 0.71, 0.76, 0.78, 0.59 for the surrogate markers: “Admitted to intensive care”; “Admitted to intermediate care”; “Admitted to hospital”; “Investigations performed in the PED” and “Uses PED resources”, respectively. The association found between triage levels and the surrogate markers of severity suggests that the PedCTAS is highly valid. Different surrogate outcome markers convey different degrees of severity, hence different degrees of urgency. Therefore, the cut-offs to calculate validation measures and the thresholds of such measures should be chosen accordingly.

## Introduction

Triage systems are inherent to the functioning of Emergency Services, establishing a hierarchy of care based on clinical risk [1, 2]. With the increasing influx of patients and frequent overcrowding [3], the adoption of a valid triage system, capable of distinguishing patients who need urgent care, from those who can wait safely, becomes paramount [4–9]. In Paediatrics, triage is an even more challenging process, due to the clinical and psychosocial characteristics of these patients and their caregivers [10, 11]. Thus, it is essential that emergency services that serve children and adolescents adopt validated models and are applied by properly trained and certified staff.

Developed in 2001 and reviewed in 2008 and 2012, Paediatric Canadian Triage and Acuity Scale (PaedCTAS) is the paediatric triage scale adopted by the Advanced Paediatric Life Support from the American Academy of Paediatrics, by the American College of Emergency Physicians (ACEP) and Canadian Association of Emergency Physicians among others [12]. It is used in several emergency services in Canada, United States and several European countries [13]. It is used in 3 out of 14 Paediatric Portuguese emergency services.

According to the ACEP and the Emergency Nurses Association (ENA), the ideal triage scale must demonstrate the characteristics of reliability, validity, utility and relevance [10]. The validity of triage systems depends on their ability to discriminate between different levels of urgency, reflecting the patient's true acuity [14].

In the absence of a gold standard for “urgency” to assess the triage’s validity, several parameters have been used, including mortality and hospitalization rates [15–17], expert opinions, Paediatric Intensive Care Unit (PICU) admissions [15, 17], length of stay [15, 16, 18], number of complementary diagnostic tests performed [17, 19], cost [19] or a combination of several indicators [20].

Several studies have evaluated the validity of the Canadian Triage and Acuity Scale(CTAS) [19, 21]. However, most of the existing studies have been conducted in Canada [22] and only a few in an European context [13]. Moreover, the studies also fail to compare the validity of surrogate outcomes markers of severity for all possible cut-offs in triage levels, needlessly aggregating information from different levels and therefore losing possibly valuable information. It is also important to assess this issue in particular regional contexts, given that the results may vary widely [13]. To the best of our knowledge is also the first PaedCTAS validity study carried out in Portugal.

Thus, we aimed to evaluate the validity of PaedCTAS triage system in a metropolitan, tertiary, university-affiliated Portuguese hospital's Paediatric Emergency Department (PED). Furthermore, we aim to study the performance and appropriateness of the different surrogate severity markers to validate triage.

## Methods

This is an observational, retrospective study that took place in PED of a metropolitan, university-affiliated hospital with a catchment area of approximately 800 thousand inhabitants, receiving approximately 76,000 visits per year from an estimated population of 137,016 children or adolescents [0–17 years] [23].

At any moment, in the PED, there are always 2 senior physicians, trained in Paediatric Emergency Medicine, 2 to 3 residents (depending on the workload), 8 nurses and 3 auxiliary staff per shift. All these teams work in 12-h shifts providing 24 h per day coverage.

The PED nursing team triages visitors from Level 1 through 5 according to the PaedCTAS [24]. To each level is assigned a different degree of urgency i.e. Level 1- “Resuscitation”, Level 2- “Emergent”, Level 3- “Urgent”, Level 4- “Less Urgent” and Level 5- “Non Urgent”, that classifies the patient based in the 3 steps: (1)the initial general state of the patient, including appearance, capillary perfusion and respiratory effort; (2)the assessment of the main complaint; and (3)the evaluation of vital signs, taking into account age and associated risk factors. Pain also plays a decisive role in this classification. Expected waiting times to be seen by a physician or reassessed are established for each priority level, i.e.level 1 are seen immediately, level 2 can wait up to 15 min, level 3 up to 30 min, level 4 up to 60 min and level 5 up to 120 min. The triage was translated to Portuguese and the translation validated with the National Emergency Nurses Association (NENA) [25]. NENA also provided training to Portuguese nurses on the PaedCTAS.

The triage itself is performed by a specialized triage nurse, and the triage level is assigned by the triage algorithm, implemented in the hospital’s information system i.e.electronic health record.

Besides having an Intensive Care Unit (ICU), this hospital also has an Intermediate Care Unit (IMCU), for patients with conditions that do not require intensive care but are also not appropriate for general admission.

In this study, all visits made by patients who were admitted the hospital’s PED (i.e.from 0 to 17 years old) in a 6-year period (between 01/Jan/2014, and 31/Dec/2019) were considered. Deceased children were excluded due to its small number and children that left without being seen and left against medical advice were excluded due to the inability to measure the surrogate outcome markers. All exclusion were reported.

For the main analysis the predictor variable used was the level assigned by the PaedCTAS. All five triage levels were split in two classes, as there are 5 levels, 4 cut-off points were used. The first cut-off considers level 1 as urgent and all the other levels non urgent i.e., 1.2345. Henceforth, this will be the nomenclature used i.e., the dot will be used to separate urgent and non-urgent levels.

The studied outcome measures, used as severity markers were:· “Admitted to intensive care”, patient’s discharge destination from the PED was the hospital’s ICU;· “Admitted to intermediate care” , patient’s discharge destination from the PED was the hospital’s IMCU;· “Admitted to hospital” patient’s discharge destination from the PED was the hospital’s inpatient care;· “Investigations performed in the PED” , which reflects the situation when a patient is asked to stay in the ED, for the physician to better assess the condition’s evolution e.g. concussion;· “Uses PED resources”, the patient is considered to “use PED resources” if during the visit the patient was medicated or if laboratory or radiologic exams were performed. The variables “Admitted to intensive care”, “Admitted to intermediate care” are subgroups of the patients “Admitted to hospital”.

For every outcome and cut-off, the sensitivity, specificity, positive predictive value(PPV), negative predictive value (NPV), positive likelihood ratio (LR +) and negative likelihood ratio (LR-) were calculated with 95% confidence intervals (CI) [26, 27]. For each outcome, Receiver Operating Characteristic (ROC) curves were plotted and the area under the curve (AUC) were calculated with 95% CI [28].

All the data analysis was performed in R version 3.4.1 (2017–06-30) [29]. The integrated development environment (IDE) used was RStudio Version 1.1.383 [30].

Ethical committee’s approval was granted for this study [FMUP 180/18].

This paper follows the structure presented in the RECORD statement i.e. The REporting of studies Conducted using Observational Routinely-collected health Data [31].

## Results

From the total of 467,917 visits to the PED within the study period, 15,119(3.23%) were removed due to the exclusion criteria and missing data, remaining 452,798(96.77%) for analysis. Deceased children(*n* = 15), patients that left without being seen (*n* = 10,658,2.28%), patients that left against medical advice (*n* = 257,0.05%) and missing data on the triage level variable (*n* = 1,859,0.40%) were removed from the analysis n.b. there is one overlap in the exclusions, therefore the sum of individual exclusions is higher than the total observations excluded.

Only one of the deceased children was not triaged level 1. The child was triaged level 2, had the first contact with the doctor 6 min later and died 17 h later, the child was transferred from another hospital and suffered from several comorbidities.

There were 1859 (0.4%) missing values in the variable “Triage Level”. Regarding the evaluated severity markers there were 4179(0.89%) missing values in the variable “admitted to hospital”, there was no missing data in the other markers.

Population characteristics are described in Table 1. There is a low variability of influx through the study years and the lower attendance during the summer. Regarding the mode of arrival of patients, most are walk-in patients, ranging from 63.1 to 92.7% for different triage levels. Most patients have home as their discharge destination, ranging from 70.1% to 93.3%, being patients triaged level 1 i.e. resuscitation, the only exception (27.5%).

**Table 1:** Summary of Paediatric Emergency Department ‘s visits’ characteristics by triage level, percentages add vertically to display the distribution within triage level

	1—Resuscitation	2—Emergent	3—Urgent	4—Less Urgent	5—Non Urgent	Overall
n	750	25888	178467	228809	18884	452798
Year (%)						
2014	101 (13.5)	4627 (17.9)	31263 (17.5)	40721 (17.8)	3091 (16.4)	79803 (17.6)
2015	121 (16.1)	4124 (15.9)	27828 (15.6)	39116 (17.1)	2975 (15.8)	74164 (16.4)
2016	150 (20.0)	4264 (16.5)	29455 (16.5)	39967 (17.5)	3748 (19.8)	77584 (17.1)
2017	152 (20.3)	4406 (17.0)	29294 (16.4)	37467 (16.4)	3629 (19.2)	74948 (16.6)
2018	118 (15.7)	4187 (16.2)	29424 (16.5)	35353 (15.5)	3078 (16.3)	72160 (15.9)
2019	108 (14.4)	4280 (16.5)	31203 (17.5)	36185 (15.8)	2363 (12.5)	74139 (16.4)
Season (%)						
Fall	200 (26.7)	7647 (29.5)	50210 (28.1)	63317 (27.7)	4622 (24.5)	125996 (27.8)
Spring	190 (25.3)	6180 (23.9)	44618 (25.0)	60548 (26.5)	4927 (26.1)	116463 (25.7)
Summer	137 (18.3)	4137 (16.0)	35183 (19.7)	47920 (20.9)	5183 (27.4)	92560 (20.4)
Winter	223 (29.7)	7924 (30.6)	48456 (27.2)	57024 (24.9)	4152 (22.0)	117779 (26.0)
Arrival time (median [IQR])	15.00 [9.00, 19.00]	16.00 [10.00, 20.00]	16.00 [11.00, 20.00]	15.00 [11.00, 19.00]	12.00 [9.00, 16.00]	15.00 [11.00, 19.00]
Sex = Male (%)	452 (60.3)	14871 (57.4)	98601 (55.2)	117511 (51.4)	9865 (52.2)	241300 (53.3)
Age (years) (median [IQR])	3.00 [1.00, 10.00]	3.00 [1.00, 11.00]	3.00 [1.00, 9.00]	8.00 [4.00, 13.00]	8.00 [3.00, 13.00]	6.00 [2.00, 12.00]
Origin (%)						
Walk-in	473 (63.1)	19200 (74.2)	140931 (79.0)	202014 (88.3)	17314 (91.7)	379932 (83.9)
Primary care provider	17 (2.3)	1640 (6.3)	10800 (6.1)	10780 (4.7)	766 (4.1)	24003 (5.3)
Healthcare call centre	5 (0.7)	1118 (4.3)	8508 (4.8)	6058 (2.6)	183 (1.0)	15872 (3.5)
Medical Emergency National Institute	166 (22.1)	1983 (7.7)	7933 (4.4)	5683 (2.5)	54 (0.3)	15819 (3.5)
Other NHS Hospital	71 (9.5)	1302 (5.0)	8172 (4.6)	3094 (1.4)	233 (1.2)	12872 (2.8)
Private Clinic	16 (2.1)	577 (2.2)	1800 (1.0)	746 (0.3)	79 (0.4)	3218 (0.7)
Outpatient consultation	2 (0.3)	41 (0.2)	116 (0.1)	109 (<0.1)	61 (0.3)	329 (0.1)
Emergency	0 (0.0)	1 (<0.1)	51 (<0.1)	59 (<0.1)	166 (0.9)	277 (0.1)
Other	0 (0.0)	14 (0.1)	22 (<0.1)	40 (<0.1)	3 (<0.1)	79 (<0.1)
(missing values in Origin)	0 (0.0)	12 (<0.1)	134 (0.1)	226 (0.1)	25 (0.1)	397 (0.1)
Discharge Destination (%)						
Home	206 (27.5)	18136 (70.1)	155144 (86.9)	213545 (93.3)	17129 (90.7)	404160 (89.3)
Primary care provider	5 (0.7)	968 (3.7)	8528 (4.8)	9255 (4.0)	562 (3.0)	19318 (4.3)
Admitted to Hospital	392 (52.3)	3562 (13.8)	8044 (4.5)	1731 (0.8)	275 (1.5)	14004 (3.1)
Outpatient consultation	28 (3.7)	763 (2.9)	3175 (1.8)	3216 (1.4)	846 (4.5)	8028 (1.8)
Other NHS Hospital	119 (15.9)	2433 (9.4)	3521 (2.0)	990 (0.4)	51 (0.3)	7114 (1.6)
Administrative discharge	0 (0.0)	5 (<0.1)	31 (<0.1)	56 (<0.1)	15 (0.1)	107 (<0.1)
Other	0 (0.0)	21 (0.1)	24 (<0.1)	16 (<0.1)	6 (<0.1)	67 (<0.1)

IQR (Inter quartile range)

The severity markers by triage level are shown in Table 2. It is important to notice the low frequency of the severity markers Admitted to intensive care and Admitted to intermediate care. It should be pointed out the increase in proportion of hospital admissions and admissions to intermediate care from level 4 to 5.

**Table 2:** Severity markers by triage level of PED visits n (%)

	1 Resuscitation	2 Emergent	3 Urgent	4 Less Urgent	5 Non Urgent	Overall
n	750	25888	178467	228809	18884	452798
Admitted to intensive care	178 (23.7)	110 (0.4)	30 (<0.1)	2 (<0.1)	2 (<0.1)	322 (0.1)
Adm. to intermediate care	76 (10.1)	1013 (3.9)	4552 (2.6)	887 (0.4)	97 (0.5)	6625 (1.5)
Adm. to hospital	511 (68.1)	6000 (23.2)	11579 (6.5)	2723 (1.2)	327 (1.7)	21140 (4.7)
Inv. performed in the PED	337 (44.9)	5228 (20.2)	7910 (4.4)	1805 (0.8)	42 (0.2)	15,322 (3.4)
Uses PED resources	689 (91.9)	22031 (85.1)	106166 (59.5)	115551 (50.5)	5928 (31.4)	250365 (55.3)

PED (Paediatric Emergency Department)

The results of the triage system’s performance as a predictor by surrogate severity markers are presented in Table 3. It shows similar trend sensitivity in all surrogate markers, lowering their values as the cut-off lowers in the triage levels. Considering the LR + the best surrogate marker was “admitted to ICU”, followed by “admitted to hospital” and “Investigations performed in the PED”. However, between the latter surrogate markers the best performance depends on the cut-off. The same is observed regarding LR-.

**Table 3:** Triage system’s validity measures by surrogate severity markers for each triage level cut-off (i.e.1.2345, 12.345, 123.45, 1234.5) in a Portuguese paediatric emergency department

Cuttoffs	1.2345	12.345	123.45	1234.5
ICU				
Sensitivity	0.55 (0.50, 0.61)	0.89 (0.86, 0.93)	0.99 (0.97, 1.00)	0.99 (0.98, 1.00)
Specificity	1.00 (1.00, 1.00)	0.94 (0.94, 0.94)	0.55 (0.55, 0.55)	0.04 (0.04, 0.04)
Positive predictive value	0.24 (0.21, 0.27)	0.01 (0.01, 0.01)	0.00 (0.00, 0.00)	0.00 (0.00, 0.00)
Negative predictive value	1.00 (1.00, 1.00)	1.00 (1.00, 1.00)	1.00 (1.00, 1.00)	1.00 (1.00, 1.00)
Positive likelihood ratio	437.28 (384.78, 496.95)	15.36 (14.77, 15.97)	2.18 (2.15, 2.21)	1.04 (1.03, 1.05)
Negative likelihood ratio	0.45 (0.40, 0.51)	0.11 (0.08, 0.15)	0.02 (0.01, 0.06)	0.15 (0.04, 0.59)
IMCU				
Sensitivity	0.01 (0.01, 0.01)	0.16 (0.16, 0.17)	0.85 (0.84, 0.86)	0.99 (0.98, 0.99)
Specificity	1.00 (1.00, 1.00)	0.94 (0.94, 0.94)	0.55 (0.55, 0.55)	0.04 (0.04, 0.04)
Positive predictive value	0.10 (0.08, 0.13)	0.04 (0.04, 0.04)	0.03 (0.03, 0.03)	0.02 (0.01, 0.02)
Negative predictive value	0.99 (0.99, 0.99)	0.99 (0.99, 0.99)	1.00 (1.00, 1.00)	0.99 (0.99, 1.00)
Positive likelihood ratio	7.59 (6.00, 9.61)	2.87 (2.72, 3.03)	1.90 (1.88, 1.92)	1.03 (1.03, 1.03)
Negative likelihood ratio	0.99 (0.99, 0.99)	0.89 (0.88, 0.90)	0.27 (0.25, 0.28)	0.35 (0.29, 0.42)
Admitted to hospital				
Sensitivity	0.02 (0.02, 0.03)	0.31 (0.30, 0.31)	0.86 (0.85, 0.86)	0.98 (0.98, 0.99)
Specificity	1.00 (1.00, 1.00)	0.95 (0.95, 0.95)	0.57 (0.57, 0.57)	0.04 (0.04, 0.04)
Positive predictive value	0.68 (0.65, 0.71)	0.24 (0.24, 0.25)	0.09 (0.09, 0.09)	0.05 (0.05, 0.05)
Negative predictive value	0.95 (0.95, 0.95)	0.97 (0.97, 0.97)	0.99 (0.99, 0.99)	0.98 (0.98, 0.98)
Positive likelihood ratio	43.66 (37.46, 50.87)	6.61 (6.45, 6.77)	1.98 (1.96, 1.99)	1.03 (1.03, 1.03)
Negative likelihood ratio	0.98 (0.97, 0.98)	0.73 (0.72, 0.73)	0.25 (0.25, 0.26)	0.36 (0.32, 0.40)
Investigations				
Sensitivity	0.02 (0.02, 0.02)	0.36 (0.36, 0.37)	0.88 (0.87, 0.88)	1.00 (1.00, 1.00)
Specificity	1.00 (1.00, 1.00)	0.95 (0.95, 0.95)	0.56 (0.56, 0.56)	0.04 (0.04, 0.04)
Positive predictive value	0.45 (0.41, 0.49)	0.21 (0.20, 0.21)	0.07 (0.06, 0.07)	0.04 (0.03, 0.04)
Negative predictive value	0.97 (0.97, 0.97)	0.98 (0.98, 0.98)	0.99 (0.99, 0.99)	1.00 (1.00, 1.00)
Positive likelihood ratio	23.30 (20.19, 26.88)	7.54 (7.36, 7.73)	2.01 (1.99, 2.02)	1.04 (1.04, 1.04)
Negative likelihood ratio	0.98 (0.98, 0.98)	0.67 (0.66, 0.68)	0.21 (0.21, 0.22)	0.06 (0.05, 0.09)
Uses Resources				
Sensitivity	0.00 (0.00, 0.00)	0.09 (0.09, 0.09)	0.51 (0.51, 0.52)	0.98 (0.98, 0.98)
Specificity	1.00 (1.00, 1.00)	0.98 (0.98, 0.98)	0.62 (0.62, 0.63)	0.06 (0.06, 0.07)
Positive predictive value	0.92 (0.90, 0.94)	0.85 (0.85, 0.86)	0.63 (0.63, 0.63)	0.56 (0.56, 0.56)
Negative predictive value	0.45 (0.45, 0.45)	0.47 (0.46, 0.47)	0.51 (0.51, 0.51)	0.69 (0.68, 0.69)
Positive likelihood ratio	9.13 (7.03, 11.87)	4.69 (4.53, 4.85)	1.37 (1.36, 1.38)	1.04 (1.04, 1.04)
Negative likelihood ratio	1.00 (1.00, 1.00)	0.93 (0.93, 0.93)	0.78 (0.77, 0.78)	0.37 (0.36, 0.38)

nb:Point estimates and 95% CIs

ICU (Intensive Care Unit), IMCU (Intermediate Care Unit)

**Fig. 1:**
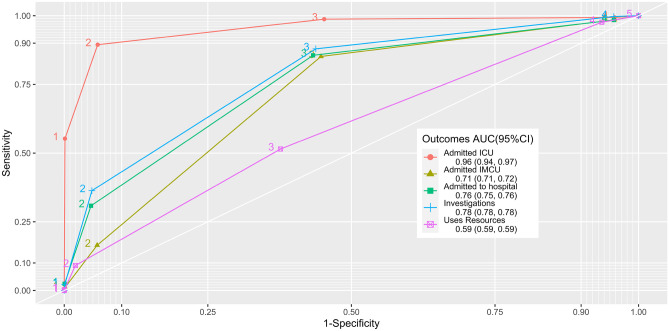
ROC curves and AUC with 95% CIs for the surrogate markers: “Admitted to intensive care”; “Admitted to intermediate care”; “Admitted to hospital”; “Investigations performed in the PED” and “Uses PED resources” in a Portuguese paediatric emergency department

Figure 1 shows the ROC curves and AUC with 95% CIs for each surrogate severity marker. It should be pointed out the good performance of the severity marker “Admitted to the ICU” and the similar performance of “admitted to hospital” and “Investigations performed in the PED”, better than “Admitted to IMCU”. Furthermore, it is important to notice the high sensitivity and specificity of the second cut-off(i.e.12.345) regarding “Admitted to the ICU”, and the similarity in sensitivity and specificity of the third cut-off for the, “Admitted to intermediate care”, “Admitted to hospital” and “Investigations performed in the PED” surrogate markers, especially in the third and fourth cut-off points.

## Discussion

The major objective of this study was to evaluate the validity of the PaedCTAS. Additionally, we studied the appropriateness of the different surrogate severity markers to validate triage.

Triage system’s validity refers to the triage system’s ability to predict ‘true’ urgency. However, the concept of ‘true’ urgency is impossible to measure since the study of the impact in delayed treatment to a patient would be unethical [32]. There are two major methodologies for triage validation:(1)those using criterion validity i.e. reference standards developed by expert panels or other triage systems; and (2)those using construct validity i.e. severity proxies [33]. In the context of diagnostics research and using surrogate markers of severity, validity can be expressed in sensitivity and specificity of a triage system, or their ratio i.e. LRs. Sensitivity represents the ability for a triage system to identify high urgent patients. Specificity represents the ability for a triage system to identify patients with low urgent problems [17]. Although there are no recommendations about the safe limits of sensitivity, under-triage or over-triage rates for emergency triage systems, an effective screening tool is expected to prioritize sensitivity, since under-triage (real high urgent patients triaged as low urgent) is unsafe [17]. On the other hand, high sensitivity may result in a low specificity. Therefore, good balance between over and under triage is important, so that “true low urgent” patients are not triaged as high urgent, compromising the flow of patients, delaying the observation of real high urgent patients and compromising their safety [34]. Going further, the balance between under-triage and over-triage i.e. sensitivity and specificity is already captured by the concept of likelihood ratios. Positive likelihood ratio answers the question: How likely is a person to be correctly triaged as urgent when compared to a person wrongly triaged as urgent? The larger the LR + , greater the likelihood of being urgent, a low value, close to one, suggests over-triage. Conversely, a negative likelihood ratio answers the question: How likely is a person be wrongly triaged as non-urgent when compared to a person correctly triaged as non-urgent? The smaller the LR-, the lesser the likelihood of being urgent. A high value, close to 1, suggests under-triage. They have a similar interpretation to PPV and NPV but without being influenced by prevalence [35].

This is the first study comparing surrogate outcomes markers of severity considering all possible triage cut-offs in the triage validation process, questioning the popular dichotomization of triage levels for performance and validity measures, this study also explores the disparity when evaluating different severity markers. It is also the first Paediatric Canadian Triage and Acuity Scale (PaedCTAS) validity study carried out in Portugal, providing groundwork to improve triage’s adequacy to the country’s population and Health Service.

The PaedCTAS performs extremely well predicting ICU admission, especially considering second cut-off(i.e.12.345). Regarding the surrogate markers “Admitted to IMCU”, “Admitted to hospital” and “Investigations performed in the PED” the results were good and very similar, particularly when assessed at the third cut-off point(i.e.123.45).

Nevertheless, given the internal PED procedures this can be explained. Patients under investigations and the IMCU are kept in the same physical space, under the same surveillance, the decision for admission in IMCU usually relates to the need for a longer observation period, and not necessarily to severity of illness.

The PaedCTAS guidelines indicate an estimated percentage for need for hospitalization for each triage level: 70% to 90% for level 1, 40% to 70% for level 2, 20% to 40% for level 3, 10% to 20% for level 4 and 0% to 10% for level 5 [36]. However our hospitalization rates were quite inferior for all triage levels: 68.1%, 23.8%, 6.5%, 1.2%, 1.7%., similar to what has been reported by several authors [16, 37, 38] and comparable to a multicentric study from Canada with 550,940 children (61%, 30%, 10%, 2%, and 0.9% for patients in Canadian Triage and Acuity Scale levels 1, 2, 3, 4, and 5, respectively) [16].

As expected, the triage system performed extremely well detecting ICU admission, these results are better than all those reported by Gravel et al. 2013 and Allon et al., and in line with Gravel et al. 2019 considering PaedCTAS. This study’s results, regarding ICU admission were better than those reported by Zachariasse et al. [22] for all triage scales.

It would be expected that the outcome “admission to IMCU” would have a performance marginally better then hospital admission, however that is not the case. This might be due to the clinical reasoning behind IMCU admission i.e. the physicians’ concern for impending clinical deterioration[, therefore IMCU admissions are less severe.

Zachariasse et al. also reviewed adult’s and children’s triage systems regarding hospital admissions n.b.in the paper the evaluation was made considering patients discharged home i.e., not admitted to hospital, therefore the values of specificity and sensibility are swapped. The results from our study are among the highest regarding sensitivity for all triage scales. The variation regarding specificity is very high among studies and our results show a low specificity, nevertheless they are very similar to other PaedCTAS studies, particularly to those with high sensitivity. These results are promising regarding external validity of this study.

Surprisingly, “investigations” i.e., when a patient is asked to stay in the ED, for the physician to better assess the condition’s evolution, performed extremely well as a triage predictor, better than hospital admissions. To the best of our knowledge, this is the first paper measuring this outcome for triage validation, hence there are no studies to compare.

“Resource use” is used in triage validation mainly using Emergency Severity Index (ESI) criteria as reference standard or having costs as an endpoint. A study showed high validity of the CTAS [21], our study had contradictory results. However, the Lee et al. study was done on elderly patients and the concept of resource utilization was CT scan and specialist consultation which contrasts with our definition i.e.if during the visit the patient was medicated or if laboratory or radiologic exams were performed. The usage of “Resource use” as a severity marker for triage validation is problematic for the lack of consensus on its concept, preventing comparisons.

Some severity markers might not be very useful detecting high urgent patients e.g., resource use and admitted to IMCU. However, changing their cut-off, they might be useful for ruling out low urgency patients.

### Limitations

Regarding the reference standard, we are aiming for is urgency, i.e., the patient condition might deteriorate quickly if he does not receive urgent care. However, we are assuming that severity equals urgency, which is not always the case, e.g., the condition of a stabilised cancer patient might be severe, and even need hospitalisation but it might not be urgent, since the patient's condition probably will not deteriorate quickly. In contrast, children with a dislocated shoulder will rarely be admitted to the hospital, although the condition needs urgent care.

This raises further questions regarding the setting variability and therefore external validity. Since the outcome might be affected by a multitude of factors [14] ranging from the quality of care to the access to the emergency department, but also local management, e.g. hospital admission's office closed after-hours and need to be admitted through the ED. In fact, this is the reason behind the unexpected increase in proportion of hospital admissions and admissions to intermediate care from level 4 to 5. Moreover, the decision of performing exams or admitting patients may have a subjective influence in the doctors, since they might take the triage level into consideration, which may have influenced the outcome [15]. Nevertheless, we believe that this is still the sound approach, for the bias can be adjusted if necessary to enable the validation comparisons, since most bias can be known by collecting data and knowledge of hospital's procedures.

Additionally, we performed our study at a single centre using a computerized version of PaedCTAS. This may limit the generalizability of the results, which would need a larger multicentre study [39].

Despite these limitations and possible variability, Hinson et al. found similar performance and validation trends across all triage scales, as well as weaknesses, hence indicating that despite these limitations it is still possible to compare triage scales and different methodologies [39].

As in most studies where a strong association was established [13, 15, 16] the frequency of hospitalization, use of PED resources, and length of stay, decreased from the higher to the lower level of urgency triage level. This study takes the validation methodology a step further, quantifying the association for each severity marker and cut-off between triage levels.

The primary operational objective of the PaedCTAS relates to how long a patient can safely wait before being seen by a physician, nonetheless the characteristics of the population, culture and the local structure of the health system, which impacts ED attendance [40, 41], plays an important role in the validation process and should be taken into account for the triage’s improvement.

### Conclusions

The association found between triage levels and the surrogate markers of severity suggests that the PedCTAS is highly valid in this context.

Different surrogate outcome markers convey different degrees of severity, hence different degrees of urgency. Therefore, the cut-offs to calculate validation measures and the thresholds of such measures should be chosen accordingly.

Likelihood ratios should be considered more often in triage validation studies. For they are more robust [27] and better convey the concepts of under and over-triage, since they incorporate information from sensitivity and specificity.

The performance of a Triage System varies greatly with the context and type of ED and ideally each PED should evaluate its own [37]. The validity of PaedCTAS is better studied in Canada and nearby countries. There are few studies evaluating the performance and validity of the PaedCTAS in a European context and none with such a thorough methodological approach. Furthermore, this study sets a baseline to evaluate future improvements to PaedCTAS.

Studies similar to this one are needed in order to improve triage systems and compare different approaches.^1^

## Data Availability

The data can be made available if requested and authorized by the hospital.
